# Functional Independence and Disability Evaluation in Stroke Patients: Optimal Cutoff Scores for a Pictorial-Based Longshi Scale, Barthel Index, and Modified Rankin Scale

**DOI:** 10.3389/fneur.2022.710852

**Published:** 2022-02-10

**Authors:** Xiangxiang Liu, Mingchao Zhou, Jingpu Zhao, Yan Gao, Yao Wang, Jing Zhou, Li Wan, Guohui Nie, Yulong Wang

**Affiliations:** ^1^Department of Rehabilitation, Shenzhen Second People's Hospital, The First Affiliated Hospital of Shenzhen University, Shenzhen, China; ^2^Department of Rehabilitation, Shenzhen Dapeng New District Nan'ao People's Hospital, Shenzhen, China

**Keywords:** stroke, functional independence, disability assessment, activities of daily living, Longshi Scale, Barthel Index, modified Rankin Scale

## Abstract

**Introduction:**

The modified Rankin Scale (mRS) and Barthel Index (BI) are widely used to measure functional outcomes worldwide. The Longshi Scale (LS), a novel pictorial-based instrument, was designed to improve the simplicity and convenience of measuring functional outcomes in the Chinese context. However, the disagreements in functional outcomes assessed by the mRS, BI, and LS are misleading, particularly in stroke patients. This study aimed to identify the optimal cutoff scores of LS and BI according to the mRS in Chinese stroke patients with different levels of functional disability.

**Methods:**

The mRS, BI, and LS were applied to evaluate functional independence and disability in 7364 stroke patients in a multi-center cross-sectional study. Stroke patients were categorized into bedridden, domestic, and community groups in advance using the LS, indicating severe, moderate, and mild functional disability, respectively. The optimal cut-off scores of the LS and BI according to the mRS were identified via sensitivity, specificity, and Youden's index and stratified by different levels of functional disability determined by LS. We also plotted the receiver operator characteristic (ROC) curves of sensitivity and specificity and determined the area under the curve (AUC).

**Results:**

In the bedridden group, LS and BI cutoff scores with the highest Youden's index were 5 and 10 for mRS 4, and the AUCs for the ROC curve were 0.848 and 0.863 for mRS 4. In the domestic group, LS and BI cutoff scores with the highest Youden's index were 5 and 65 for mRS 3, and the AUCs for the ROC curve were 0.796 and 0.826 for mRS 3. In the community group, LS cutoff scores with the highest sum of sensitivity and specificity were 9, 9, and 8 for mRS grades 0, 1, and 2, respectively, while the BI cutoff scores with the highest sum of sensitivity and specificity were 100, 100, and 95, respectively, while the AUCs for the ROC curve were 0.697 and 0.735 for mRS 2, 0.694 and 0.716 for mRS 1, and 0.628, and 0.660 for mRS 0.

**Conclusions:**

The mRS is more precise to determine mild functional disability, whereas BI can provide more specific information on moderate and severe levels in stroke patients. Although LS was a less precise was to determine moderate and severe levels than BI, it is much simpler and more convenient to be applied to a large-scale population.

## Introduction

Stroke leads to a range of unfavorable functional outcomes (1). Improving the post-stroke disability and functional independence has been the core rehabilitation content among stroke patients (2). Appropriate assessment of functional outcomes is beneficial to provide individualized rehabilitation treatment and evaluate the corresponding therapeutic effect (2). The modified Rankin Scale (mRS) and Barthel Index (BI) are the most widely used assessment tools for functional outcomes in stroke patients (3).

The mRS defines seven grades of functional disability, from 0 indicating “no symptoms at all” to 6 indicating “death” (4, 5). The BI consists of 10 items, including bowel control, bladder control, grooming, toilet use, feeding, transfers (bed to chair and back), mobility (on level surfaces), dressing, stairs, and bathing (6). Accumulative studies have indicated that the mRS and BI had excellent reliability, adequate validity, and responsiveness, which are recommended as the primary tools to explain the functional outcome of stroke survivors (6–9). However, some limitations of the mRS and BI quantified when implemented in the clinical rehabilitation field have been identified. For example, a previous systematic review indicated that the BI score is linear ranging from 0–100, its subtle fluctuation is not of clinical significance and is difficult to interpret (7, 10). Additionally, the mRS and BI focus on the assessment of body function and activity; however, they are limited in assessing family and social participation ability (11), which is one of three components in the international framework for classifying functional disability (12). These findings suggest that the functional assessment scales considering participation ability are necessary.

The LS, a novel pictorial-based tool for assessing functional outcomes in the Chinese context, was developed based on a survey involving 1,862 patients with physical disabilities in 2013 (13). It consists of 27 pictorial-based items and can appropriately categorize participants into bedridden, domestic, and community groups. Importantly, the LS extended to consider the functions of family and social participation in the aspect of assessment items. Similarly, the LS has shown good validity and reliability in a psychometric properties survey involving 869 inpatients with functional disability (13). The LS is now recommended as one of the Chinese National Standards (license code: GB/T 37103-2018) to assess functional independence and disability (14).

The difference in assessing functional outcomes via the mRS, BI, and LS remains unclear, particularly in Chinese stroke patients. A previous study involving 5,475 acute stroke patients has indicated that the LS was significantly associated with the mRS and BI; however, participants did not include patients with other stroke subtypes, including subacute and chronic stroke (12). Moreover, the previous study did not consider the potential ceiling effect or floor effect of these scales, which might lead to insensitive evaluations in stroke patients with different severities. The purpose of this study was to investigate the relationship of mRS, BI, and LS, and to determine the optimal cutoff of LS and BI scores according to mRS grades in stroke patients with different durations and different severities.

## Materials and Methods

### Study Design

This study was a multi-center and cross-sectional study.

### Setting

This study was conducted in the departments of rehabilitation medicine of 103 hospitals located in 23 cities of China from September 2018 to April 2020.

### Participants

Originally, the study recruited 15,205 consecutive inpatients from the Department of Rehabilitation Medicine. Of them, 7,364 stroke patients with a duration from 1 to 45 months were selected for further analysis. The diagnosis of stroke was based on the 10 th revision of the International Classification of Diseases (I60.x and I61.x for the hemorrhagic subtype; H34.1, I63.x, and I64.x for the ischemic subtype) (15). Stroke patients who could not read and answer questions and suffered from deformities, mental illnesses, aphasia, and cognitive dysfunction were excluded. Furthermore, stroke patients with a Mini-Mental State Examination (MMSE) score <27 were also excluded (16). The potential reason is that patients with MMSE scores <27 have difficulty in recognizing the pictorial-based items of the LS. Additionally, patients who participated in other clinical studies simultaneously were excluded.

### Data Collection

Questionnaires were used to collect patients' sociodemographic data. Functional independence and disability were assessed by registered physicians or therapists using the Quicker Recovery Line (QRL) platform (17), a rehabilitation evaluation and management system. First, a professional logged in to the QRL application and asked patients to sign an informed consent form. Second, the LS, BI, and mRS were randomly assigned to physicians and therapists based on the QRL system. Lastly, the physicians and therapists received the assigned scales and were required to finish the assessment tasks within 15 days. LS, BI, and mRS were evaluated by interviewing patients face-to-face. All data were recorded on electronic forms and uploaded to the QRL online system. Missing information was handled by requesting that the practitioner re-interview the participant.

### Outcome Measurement

LS was a pictorial-based scale originally designed by the Rehabilitation Department of Shenzhen Second People's Hospital to assess activities of daily living (ADL) in patients with functional disabilities. The LS measurement protocol has been previously outlined in detail (13). The LS assessment consisted of the following steps: first, all participants were assigned to the bedridden, domestic, or community groups depending on their ability to get out of the bed, go outdoors, and return indoors. Individuals in the bedridden group were defined as one who is unable to get out of bed independently. Individuals in the domestic group were defined as one who is able to get out of the bed independently but unable to go outdoors independently with or without an assistive device. Individuals in the community group were defined as one who can go outdoors independently with or without an assistive device. The categorization was based on the patient's transfer ability and mobility scope, rather than the ability to perform ADL; second, patients in each group were evaluated using a 3-point Likert subscale, specifically: (1) bedridden group subscale (including bladder and bowel management, feeding, and leisure activities); (2) domestic group subscale (including toileting, grooming, and housework), and (3) community group subscale (including community mobility, shopping, and social participation). Finally, the total score of each subscale was calculated (13).

The BI includes 10 aspects of ADL and shows high reliability and validity in the Chinese population: (1) feeding; (2) moving from wheelchair to the bed and back to wheelchair; (3) personal toilet; (4) getting on and off the toilet; (5) self-bathing; (6) walking on level surface; (7) ascending and descending stairs; 8) dressing; (9) controlling bowel movements; and (10) bladder control. Items 8 and 9 included four levels and were marked as 15, 10, 5, and 0 based on the independence of ADL: items 1, 4–7, and 10 included three levels and were marked as 10, 5, and 0, while items 2 and 3 only included two levels and were marked as 5 and 0, respectively. The total score ranged 0-100, with a higher score indicating a higher functional ability (18).

The mRS defined six different grades of disability from 0 (no symptoms) to 6 (death); however, in this study, mRS 6 was not included, because dead patients were unavailable to undergo LS and BI evaluation. All scales were assessed by interviewing patients face-to-face (3).

### Data Analysis

Statistical analyses were conducted using SPSS (version 22.0; IBM Corp., Armonk, NY, USA). Demographic characteristics are presented as numbers (%). Demographic characteristics included age, gender, ethnicity (Han and Minority), marital status (married, unmarried, divorced, and widowed), living status (living alone, living with family, living with tender, living in nursing institution, and other), annual household income (−50,000, 50,000–100,000, 100,000–150,000, and >150,000 *yuan*), education level (primary and lower, high school, college, and higher), hospital style (provincial, prefecture, country, and community-level), and hospital level (primary, secondary, and tertiary). Religion and retirement were coded as “yes” and “no”. All data analyses were performed separately in bedridden, domestic, and community groups. Since the LS is a classification scale, it contains three 3-point Likert subscales, which are suitable for assessing severe, moderate, and mild impairment, respectively.

Boxplots were constructed to show the distribution of BI, LS, and mRS scores in the three groups. Sensitivity, specificity, and Youden's index of LS and BI scores for the mRS grades were calculated. The sensitivity and specificity of a scale depend on the level chosen as the cutoff point for favorable (negative or normal) and unfavorable (positive or abnormal) conditions (19). In this study, outcomes were divided into favorable and unfavorable groups using different mRS grades to obtain the corresponding LS and BI scores. For example, to calculate corresponding BI scores for mRS grade 1, mRS 0 or 1 were set as favorable outcomes, and mRS 2 to 5 were set as unfavorable outcomes. Sensitivity was defined as the proportion of cases that were greater or equal to the LS or BI cutoff score among favorable outcomes. Specificity was defined as the proportion of cases that were below the LS or BI cutoff scores among unfavorable outcomes. Youden's index was defined as the sum of sensitivity and specificity −1.

The curve of receiver operating characteristic (ROC) is widely accepted as a method for selecting an optimal cutoff point for a scale and for comparing the accuracy of different scales (12, 19, 20). The curve was generated by plotting the sensitivity of all possible cutoff points for the scale on the y-axis as a function of 1-specificity on the x-axis. The area under the ROC curve provides a measure of the overall performance of a scale. The area under the curve (AUC) reflects the extent to which the scale distinguishes between favorable and unfavorable outcomes. The AUC serves as a single measure, independent of prevalence, that summarizes the discrimination ability of a scale across the full range of cutoffs. The greater the AUC, the better the scale. A perfect scale will have an AUC of 1.0, while a completely inadequate scale (one whose curve falls on the diagonal line) has an AUC of 0.5 (19).

## Results

### Characteristics of Participants

**Figure 1:**
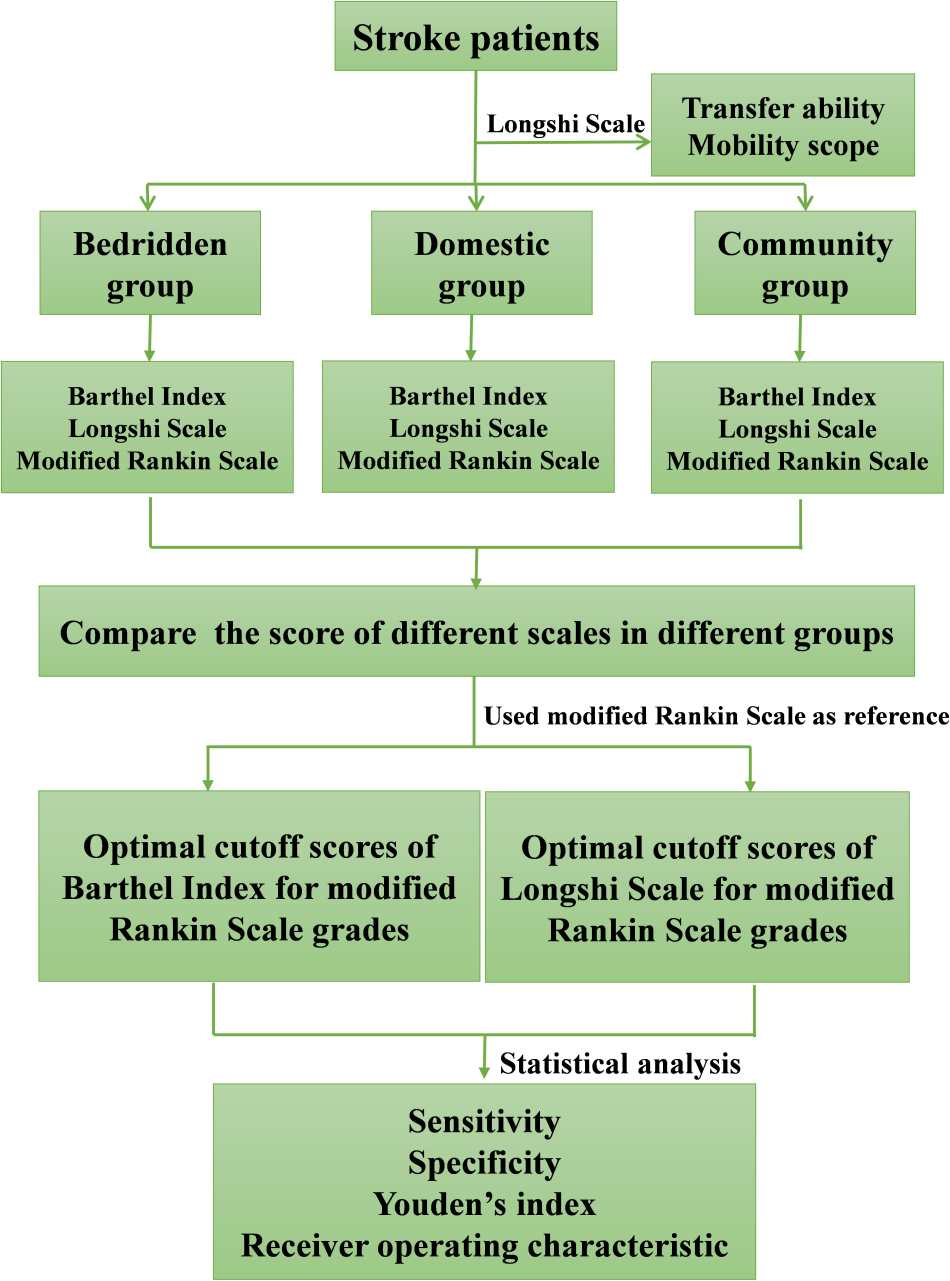
Flow chart of study process.

A total of 7364 stroke patients aged 67.4 ± 14.48 years were included in this study. The study process is illustrated in Figure 1. Participants' characteristics in the bedridden, domestic, and community groups are summarized in Table 1. The majority of subjects were men (*n* = 4,457, 60.5%), Han ethnicity (*n* = 7,345, 99.7%), had a high school educational level (*n* = 5,994, 81.4%), were non-religious (*n* = 6,911, 92.5%), and lived with family (*n* = 5,347, 72.6%). Among all the participants, 4,079 (55.4%), 2,082 (28.3%), and 1,203 (16.3%) stroke patients were classified into the bedridden, domestic, and community groups, respectively. In the bedridden group, 23 (0.3%), 22 (0.3%), 93 (1.3%), 131 (1.8%), 2,184 (29.7%), and 1,626 (22.1%) participants scored 0, 1, 2, 3, 4, and 5, respectively. In the domestic group, 15 (0.2%), 123 (1.7%), 285 (3.9%), 787 (10.7%), 782 (10.6%), and 90 (4.3%) participants had mRS scores of 0, 1, 2, 3, 4, and 5, respectively. In the community group, 63 (0.9%), 484 (6.6%), 334 (4.5%), 215 (2.9%), 51 (0.7%), and 56 (0.8%) participants scored 0, 1, 2, 3, 4, and 5, respectively.

**Table 1:** The sociodemographic characteristics of surveyed stroke patients.

**Items**	**Category**	**Bedridden** **group**		**Domestic** **group**		**Community** **group**	
		**n**	**%**	**n**	**%**	**n**	**%**
**Gender**	Male	2,319	31.5	1,350	18.3	788	10.7
	Female	1,760	23.9	732	9.9	415	5.6
**Ethnicity**	Han	4,607	55.2	2,076	28.2	1202	16.3
	Minority	12	0.2	6	0.1	1	0.0
**Marriage**	Married	3,569	48.5	1,852	25.1	1108	15.0
	Unmarried	47	0.6	40	0.5	28	0.4
	Divorce	52	0.7	36	0.5	17	0.2
	Widowed	411	5.6	154	2.1	50	0.7
**Living status**	Living alone	153	2.1	82	1.1	49	0.7
	Living with family	2,723	37.0	1,576	21.4	1048	14.2
	Living with tender	415	5.6	129	1.8	22	0.3
	Living in nursing institution	306	4.2	106	1.4	35	0.5
	other	482	6.5	189	2.6	49	0.7
**Religion**	Yes	311	4.2	175	2.4	67	0.9
	No	3,768	51.2	1,907	25.9	1136	15.4
**Education level**	Primary school and below	1277	17.3	590	8.0	295	4.0
	High school	2,121	28.8	1,080	14.7	631	8.6
	College	473	6.4	329	4.5	242	3.3
	Graduate school	208	2.8	83	1.1	35	0.5
**Retirement**	Yes	2,902	39.4	1,314	17.8	663	9.0
	No	1,177	16.0	768	10.4	540	7.3
**Family annual income**	Less than ¥50 thousands	1,227	16.7	704	9.6	356	4.8
	¥50–100 thousands	1,661	22.6	785	10.7	428	5.8
	¥100–150 thousands	703	9.5	354	4.8	245	3.3
	¥150–200 thousands	280	3.8	125	1.7	101	1.4
	More than ¥200 thousands	208	2.8	114	1.5	73	1.0
**Style of hospital**	Provincial-level	1,066	14.5	577	7.8	397	5.4
	Prefecture-level	2,066	28.1	993	13.5	514	7.0
	Country-level	362	4.9	290	3.9	228	3.1
	Community-level	585	7.9	222	3.0	64	0.9
**Level of hospital**	Primary	2,530	34.4	1,279	17.4	850	11.5
	Second	1,189	16.1	684	9.3	308	4.2
	Tertiary	360	4.9	119	1.6	45	0.6
**mRS**	0	23	0.3	15	0.2	63	0.9
	1	22	0.3	123	1.7	484	6.6
	2	93	1.3	285	3.9	334	4.5
	3	131	1.8	787	10.7	215	2.9
	4	2,184	29.7	782	10.6	51	0.7
	5	1,626	22.1	90	1.2	56	0.8
**Total**		4,079	55.4	2,082	28.3	1203	16.3

### Scores of LS, BI, and mRS in Different Groups

**Figure 2:**
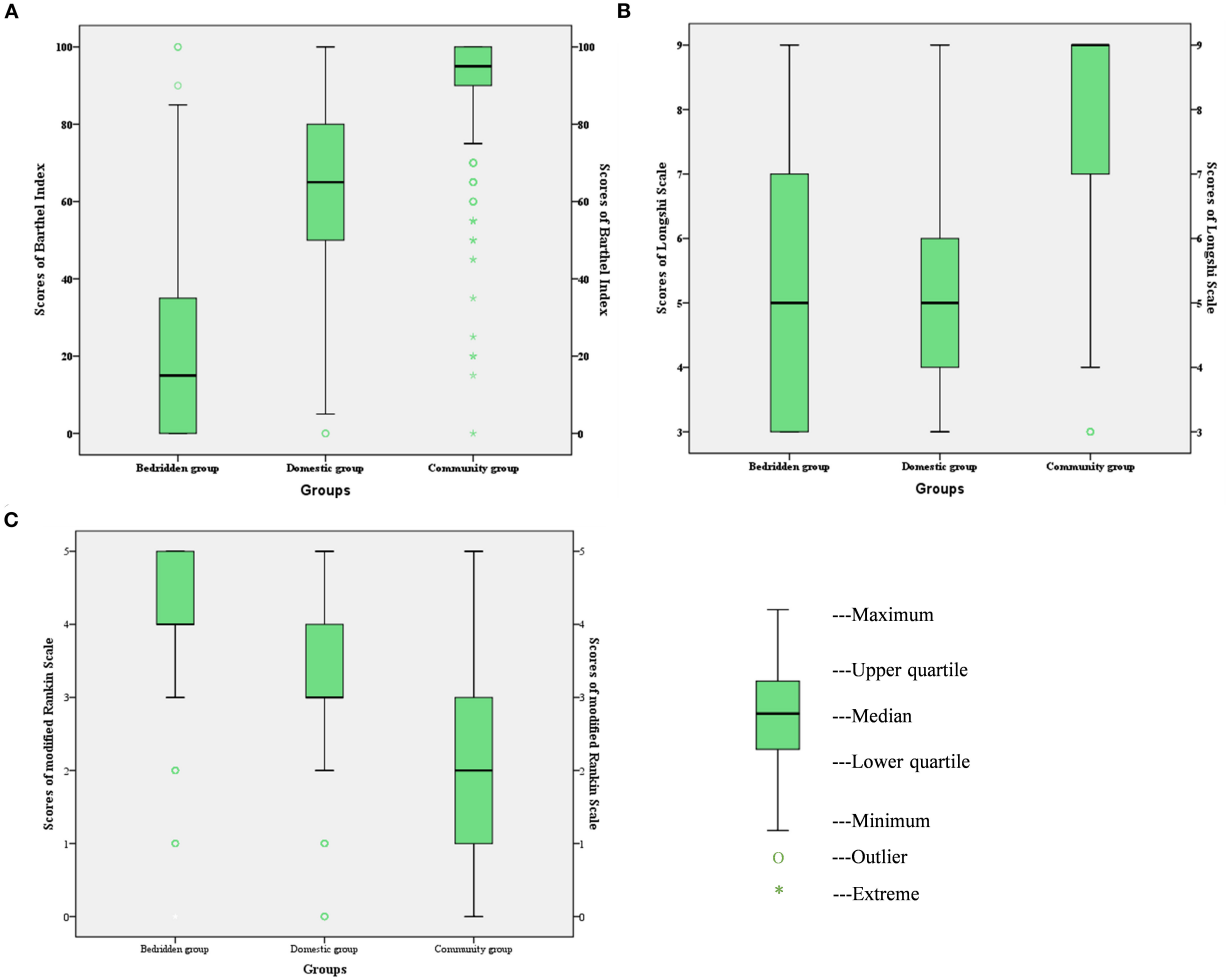
Distribution of BI scores **(A)**, LS scores **(B)**, and mRS scores **(C)** in the bedridden group, domestic group and community group. BI, Barthel Index; LS, Longshi Scale; mRS, modified Rankin Scale.

The distributions of LS, BI, and mRS scores in the bedridden, domestic, and community groups are presented as boxplots in Figure 2. For BI, the score range was wide in the bedridden and domestic groups, and varied between 0 and 85, and 0 and 100, respectively (Figure 2A). Similarly, for LS, the score range was wide in these groups, and varied between 0 and 9 (Figure 2B). In contrast, the range of mRS scores was wide in the community group and varied between 0 and 5 (Figure 2C).

### Sensitivity and Specificity of LS and BI Corresponding to mRS Grades

The sensitivity, specificity, and Youden's index for the cutoff scores of LS and BI in relation to mRS grades were calculated (see Table 2). For mRS 0, the optimal cutoff score for BI was 100, with a sensitivity and specificity of 74.6% and 52.6%, respectively. For mRS 1, the BI score with the highest sum of sensitivity and specificity was 100, with a sensitivity and specificity of 68.9% and 68.0%, respectively. For mRS 2, the BI score with the highest sum of sensitivity and specificity was 95, with a sensitivity of 76.7% and a specificity of 62.1%. For mRS 3, the BI score with the highest sum of sensitivity and specificity was 65, with a sensitivity of 76.9% and a specificity of 73.5%. For mRS 4, the BI score with the highest sum of sensitivity and specificity was 10, with a sensitivity of 85.1% and a specificity of 78.1%.

**Table 2:** Sensitivity, specificity, and Youden's index for LS and BI cutoff scores corresponding to mRS grades*.

**Groups**	**Grades**	**LS**				**BI**			
		**Scores**	**Sensitivity**	**Specificity**	**Youden's index**	**Scores**	**Sensitivity**	**Specificity**	**Youden's index**
Community group	mRS 0	9	0.794	0.417	0.211	100	0.746	0.512	0.272
	mRS 1	9	0.782	0.562	0.344	100	0.689	0.680	0.369
	mRS 2	8	0.778	0.438	0.216	95	0.767	0.621	0.388
Domestic group	mRS 3	5	0.740	0.747	0.484	65	0.769	0.735	0.504
Bedridden group	mRS 4	5	0.797	0.818	0.615	10	0.851	0.781	0.632

*Youden's index = (Sensitivity + Specificity)-1. LS, Longshi Scale; BI, Barthel Index; mRS, modified Rankin Scale.

For mRS 0, the optimal cutoff score for LS was 9, with a sensitivity of 79.4% and a specificity of 41.7%. For mRS 1, the LS score with the highest sum of sensitivity and specificity was 9, with a sensitivity of 78.2% and a specificity of 56.2%. For mRS 2, the LS score with the highest sum of sensitivity and specificity was 8, with a sensitivity of 77.8% and a specificity of 43.8%. For mRS 3, the LS score with the highest sum of sensitivity and specificity was 5, with a sensitivity of 74.0% and a specificity of 74.7%. For mRS 4, the LS score with the highest sum of sensitivity and specificity was 5, with a sensitivity of 79.7% and a specificity of 81.8%.

From the results, we found that the score range and Youden's index increased by mRS grades both in LS and BI, which indicated that LS and BI sensitively differentiated moderate and severe patients, while the mRS was better for assessing mild functional disability in stroke patients.

### Optimal Cutoff Scores of LS and BI Corresponding to mRS Grades

**Figure 3:**
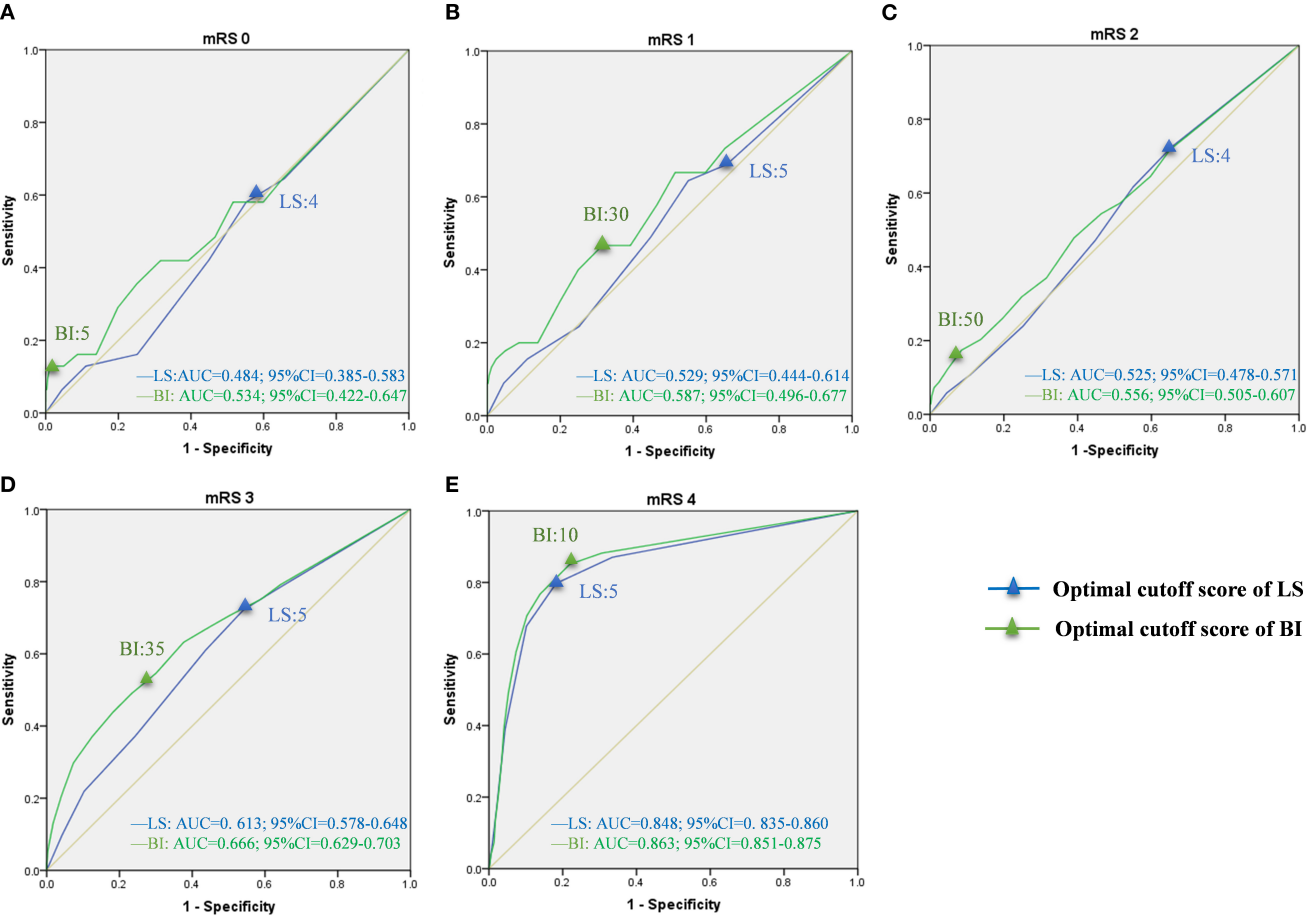
ROC curves of LS and BI cutoff scores corresponding to mRS grades in the bedridden group. LS, Longshi Scale; BI, Barthel Index; mRS, modified Rankin Scale; ROC, Receiver Operating Characteristic; AUC, Area Under Curve; CI, Confidence Interval. **(A)** ROC curves for LS and BI cutoff scores in mRS 0. Optimal cutoff score: 4 and 5; Sensitivity: 64.5 and 64.5%; specificity: 34.3 and 34.7%; AUC: 0.484 and 0.534, respectively. **(B)** ROC curves for LS and BI cutoff scores in mRS 1. Optimal cutoff score: 4 and 30; Sensitivity: 64.4 and 46.7%; specificity: 44.9 and 68.5%; AUC: 0.529 and 0.587, respectively. **(C)** ROC curves for LS and BI cutoff scores in mRS 2. Optimal cutoff score: 4 and 50; Sensitivity: 72.5 and 17.4%; specificity: 34.6 and 91.5%; AUC: 0.525 and 0.556, respectively. **(D)** ROC curves for LS and BI cutoff scores in mRS 3. Optimal cutoff score: 5 and 35; Sensitivity: 72.1 and 49.1%; specificity: 46.0 and 76.6%; AUC: 0.613 and 0.666, respectively. **(E)** ROC curves for LS and BI cutoff scores in mRS 4. Optimal cutoff score: 5 and 10; Sensitivity: 79.7 and 85.1%; specificity: 81.8 and 78.1%; AUC: 0.848 and 0.863, respectively.

**Figure 4:**
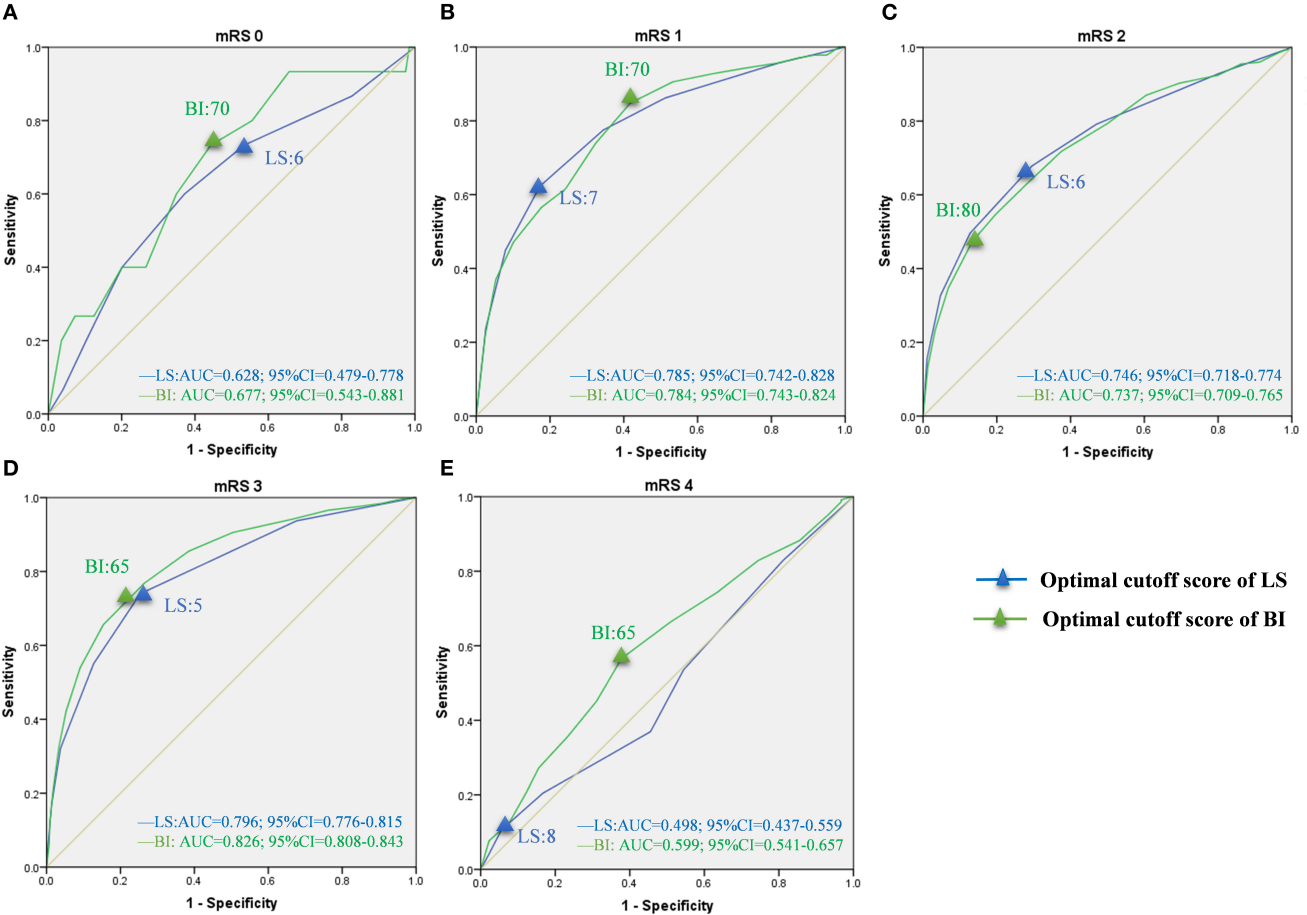
ROC curves of LS and BI cutoff scores corresponding to mRS grades in the domestic group. LS, Longshi Scale; BI, Barthel Index Scale; mRS, modified Rankin Scale; ROC, Receiver Operating Characteristic; AUC, Area Under Curve; CI, Confidence Interval. **(A)** ROC curves for LS and BI cutoff scores in mRS 0. Optimal cutoff score: 6 and 70; Sensitivity: 60.0 and 73.3%; specificity: 62.8 and 55.6%; AUC: 0.628 and 0.677, respectively. **(B)** ROC curves for LS and BI cutoff scores in mRS 1. Optimal cutoff score: 7 and 70; Sensitivity: 62.3 and 84.8%; specificity: 82.7 and 58.3%; AUC: 0.785 and 0.784, respectively. **(C)** ROC curves for LS and BI cutoff scores in mRS 2. Optimal cutoff score: 6 and 80; Sensitivity: 67.8 and 54.6%; specificity: 70.5% and 80.4%; AUC: 0.746 and 0.737, respectively. **(D)** ROC curves for LS and BI cutoff scores in mRS 3. Optimal cutoff score: 5 and 65; Sensitivity: 74.0 and 76.9%; specificity: 74.7 and 73.5%; AUC: 0.796 and 0.826, respectively. **(E)** ROC curves for LS and BI cutoff scores in mRS 4. Optimal cutoff score: 8 and 65; Sensitivity: 10.6 and 94.4%; specificity: 56.6 and 62.2%; AUC: 0.498 and 0.599, respectively.

**Figure 5:**
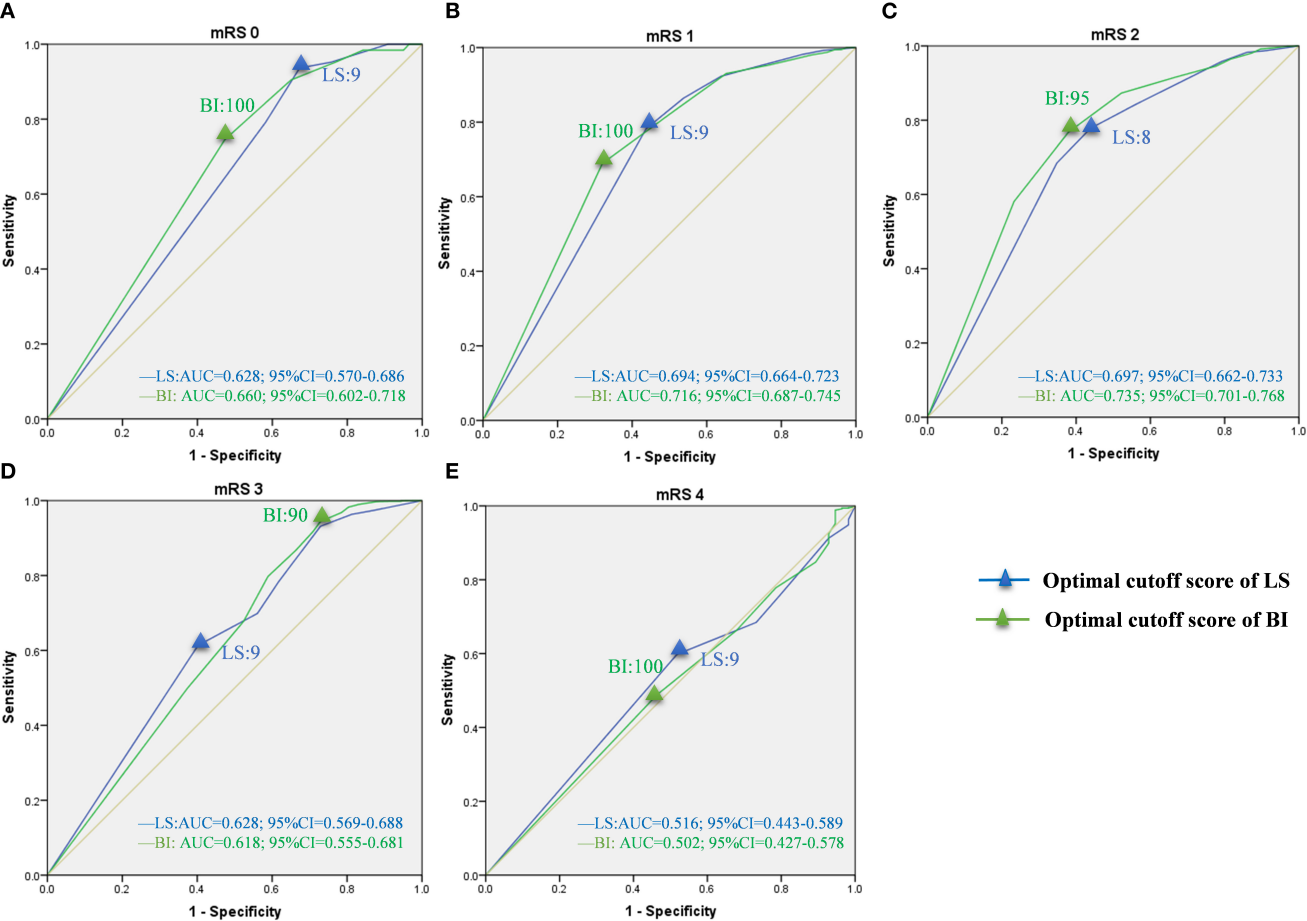
ROC curves of LS and BI cutoff scores corresponding to mRS grades in the community group. LS, Longshi Scale; BI, Barthel Index Scale; mRS, modified Rankin Scale; ROC, Receiver Operating Characteristic; AUC, Area Under Curve; CI, Confidence Interval. **(A)** ROC curves for LS and BI cutoff scores in mRS 0. Optimal cutoff score: 9 and 100; Sensitivity: 93.7 and 74.6%; specificity: 32.7 and 52.6%; AUC: 0.628 and 0.660, respectively. **(B)** ROC curves for LS and BI cutoff scores in mRS 1. Optimal cutoff score: 9 and 100; Sensitivity: 78.2 and 68.9%; specificity: 56.2 and 68.0%; AUC: 0.694 and 0.716, respectively. **(C)** ROC curves for LS and BI cutoff scores in mRS 2. Optimal cutoff score: 8 and 95; Sensitivity: 77.8 and 76.7%; specificity: 43.8 and 62.1%; AUC: 0.697 and 0.735, respectively. **(D)** ROC curves for LS and BI cutoff scores in mRS 3. Optimal cutoff score: 9 and 75; Sensitivity: 61.3 and 94.4%; specificity: 59.8 and 27.1%; AUC: 0.628 and 0.618, respectively. **(E)** ROC curves for LS and BI cutoff scores in mRS 4. Optimal cutoff score: 9 and 100; Sensitivity: 59.8 and 48.9%; specificity: 48.2 and 53.6%; AUC: 0.516 and 0.502, respectively.

Based on sensitivity and specificity, the AUCs were computed for LS and BI in the bedridden, domestic, and community groups (Table 3). The curves for each group are presented in Figures 3–5. In the bedridden group, the maximal AUCs for the LS and BI cutoff scores were 0.848 (95% CI, 0.835–0.860) and 0.863 (95% CI, 0.851–0.875) in mRS 4 (see Figure 3E). In the domestic group, the maximal AUCs for the LS and BI cutoff scores were 0.796 (95% CI, 0.776–0.815) and 0.826 (95% CI, 0.808–0.843) in mRS 3 (see Figure 4D). In the community group, the maximal AUCs for the LS and BI cutoff scores were 0.697 (95% CI, 0.663–0.733) and 0.735 (95% CI, 0.701–0.768) in mRS 4 (Figure 5C). Based on the results, we recommend using these cutoff scores to define and interpret outcomes in stroke patients with different levels of severity.

**Table 3:** Area under the curve (AUC) of the LS and BI cutoff scores corresponding to mRS grades^*^.

**Items**		**Bedridden group**			**Domestic group**			**Community group**		
**Grades**	**Scales**	**Area**	**Asymptotic 95% confidence interval**		**Area**	**Asymptotic 95% confidence interval**		**Area**	**Asymptotic 95% confidence interval**	
			**Lower bound**	**Upper bound**		**Lower bound**	**Upper bound**		**Lower bound**	**Upper bound**
mRS 0	LS	0.460	0.333	0.588	0.628	0.479	0.778	0.628	0.570	0.686
	BI	0.544	0.391	0.647	0.689	0.543	0.811	0.660	0.602	0.718
mRS 1	LS	0.529	0.444	0.614	0.785	0.742	0.828	0.694	0.664	0.723
	BI	0.587	0.496	0.677	0.784	0.743	0.824	0.716	0.687	0.745
mRS 2	LS	0.525	0.478	0.571	0.746	0.718	0.774	0.697	0.662	0.733
	BI	0.556	0.505	0.607	0.737	0.709	0.765	0.735	0.701	0.768
mRS 3	LS	0.613	0.578	0.648	0.796	0.776	0.815	0.628	0.569	0.688
	BI	0.666	0.629	0.703	0.826	0.808	0.843	0.618	0.555	0.681
mRS 4	LS	0.848	0.835	0.860	0.498	0.437	0.559	0.516	0.443	0.589
	BI	0.863	0.851	0.875	0.599	0.541	0.657	0.502	0.427	0.578

* *LS, Longshi Scale; BI, Barthel Index; mRS, modified Rankin Scale*.

## Discussion

In this study, we divided stroke patients into three groups based on their transfer ability and mobility scope. Then, we analyzed the optimal cutoff scores for LS and BI corresponding to mRS grades in different groups. In the bedridden group, LS and BI cutoff scores were 5 and 10 for the mRS 4. In the domestic group, LS and BI cutoff scores were 5 and 65 for the mRS 3. In the community group, LS cutoff scores were 9, 9, and 8 for mRS grades 0, 1, and 2, respectively, while BI cutoff scores were 100, 100, and 95, respectively. These results may help clinicians in designing clinical trials, and interpreting treatment outcomes in stroke trials. For instance, the change in LS, BI, and mRS categories indicated clinically meaningful improvement in ADL in stroke patients (2). Thus, academics can estimate whether the score change in a group is associated with the intervention effect.

The mRS and BI have been compared extensively in stroke trials. However, there is little consensus on the optimal implementation of the BI and mRS as outcome measure in stroke trials. Most existing stroke trials have defined favorable outcomes (negative or normal) with mRS ≦ 2 (2, 21, 22). Hacke et al. have reported that mRS ≦ 2 and BI ≧ 75 could be used to determine favorable or unfavorable outcomes of stroke trials (sensitivity: 75.0%; specificity: 97.8%) (23). However, Maarten et al. found mRS ≦ 2 and BI ≧ 90 to be the optimal cutoff score to determine favorable or unfavorable outcome (sensitivity: 90.7%, specificity: 88.1%) (24). Another study of 5,759 stroke patients in Korea found the optimal cutoff scores of the modified BI to be 98 (sensitivity: 90.4%; specificity 83.8%) and 94 (sensitivity: 88.5%; specificity: 93.7%) for mRS 1 and 2, respectively (2). Our previous study found the optimal cutoff points of BI to be 100 (sensitivity: 100%; specificity 95.3%) and 100 (sensitivity: 98.1%; specificity 100%) corresponding to the adjusted mRS 1 and 2, respectively (4). Based on this study, mRS grade 2 was classified as the community group, and the corresponding cutoff scores were LS ≧ 8 and BI ≧ 95; therefore, community groups may be used with corresponding cutoff scores of LS and BI to determine favorable or unfavorable outcomes of stroke trials.

In this study, we found that LS and BI cutoff score ranges were variable for each mRS grade. The mRS grades 0, 1, and 2 exhibited a narrow range of LS and BI scores, while mRS grades 3 and 4 demonstrated a broad score range. The LS cutoff scores for mRS 0, 1, and 2 were 9, 9 and 8. The BI cutoff scores were 100, 100, and 95 for mRS scores of 0, 1, and 2, respectively. These cutoff scores were near the maximum, indicating the ceiling effect of LS and BI. The ceiling effect is commonly observed in BI (25). However, this is the first report of ceiling effects in LS. In reality, both LS and BI focus on basic ADLs and lack information on many instrumental ADLs. Thus, many tasks not measured by LS or BI might play crucial roles in disability after stroke. The BI score 100 or LS score 9 do not mean that a patient is able to perform advanced ADLs independently. These results suggest that both LS and BI may not be sensitive tools for assessing functional independence and disability in patients with mild stroke.

The instruments for distinguishing outcomes with better and worse screening values are also vital interpretation criteria. Based on our results, we found that BI had higher sensitivity, specificity, and AUCs than LS, corresponding to mRS grades. BI reflected better validity and reliability than LS in stroke patients, which could be available for clinical diagnosis and intervention evaluation in stroke trials. However, LS is simple, time-saving, and easy to understand with vivid pictures, and is more suitable to screen patients with moderate or severe functional disabilities on a large scale. Moreover, a previous study showed that LS could be used by non-professionals who had minimal training to assess ADLs of functionally disabled patients (13). Considering the characteristics of LS, it can be used for disability screening in rural areas with insufficient healthcare professionals.

In this study, we used the mRS as a reference to dichotomize the LS and BI, which were used to assess functional independence and disability. LS is a novel, picture-based scale that has been developed and widely used in China (26). Although the LS is simple, easy to use, and has good inter-rater and intra-rater reliability (ICC > 0.8) (13), it is not used globally. The BI is an ordinal and non-continuous scale with a larger score range, facilitating the selection of cutoff scores compared with mRS (27). The mRS is a clinically relevant scale, with six different clear and well-defined grades. It is also proven to have acceptable reliability and is correlated with the BI (4). Compared with BI, mRS has fewer variations in stroke trials (28, 29). Thus, it is suitable to use mRS cutoff scores as a reference to distinguish between favorable and unfavorable outcomes.

Our study has the following limitations. The sampling method was non-random and included hospitals that collaborated with our departments. However, the larger sample size from multi-centers might decrease the bias caused by the non-random sampling methods. Also, we only selected stroke patients in the Chinese population; therefore, our results may not be applicable to other national populations. Data was collected by the online system and required estimators to finish assessment tasks within 15 days and this increased interval may result in function change. Thus, we invited experienced investigators to assess LS, BI, and mRS randomly to reduce measurement bias as much as possible. Additionally, our study included both first and recurrent stroke patients, and it may not be possible to differentiate between pre- or post-stroke disability or to standardize the frequency or duration of stroke incidence, which might affect the assessment results. Thus, long-term prospective studies are needed to identify the differences between these aspects to provide improved intervention strategies. Finally, patients with mental illnesses, cognitive dysfunction, and aphasia were excluded from the study, since they had difficulty in completing the assessment, which could lead to a selection bias. In the future, we plan to explore the consistency between the assessment results of caregivers and patients to expand the application scenarios of these scales.

## Conclusions

The measurement capacities of LS, BI, and mRS for measuring the functional outcomes in stroke patients are different. The mRS is more sensitive in differentiating mild disability among stroke survivors, while LS and BI are more sensitive in differentiating moderate and severe stroke patients. LS is simpler and more concise, but less precise, than BI, and it seemed to be suitable for screening patients with moderate and severe functional disability. The BI can provide more specific information about ADLs, which are available for clinical diagnosis and intervention evaluation in stroke trials.

## Data Availability Statement

The raw data supporting the conclusions of this article will be made available by the authors, without undue reservation.

## Ethics Statement

The studies involving human participants were reviewed and approved by Medical Ethics Committees of the First Affiliated Hospital of Shenzhen University. The patients/participants provided their written informed consent to participate in this study.

## Author Contributions

XL and YuW: conceptualization. XL and MZ: methodology. JZha:software. YaW, JZho, and MZ: validation and investigation. XL, MZ, and LW: formal analysis. YuW: resources. LW: data curation. XL: writing—original draft preparation. JZha and YG: writing—review and editing. JZha: visualization. GN: supervision. YuW: project administration. All authors approved the final version of the manuscript for submission.

## Funding

YuW, JZho and XL were supported by the National Key R&D Program of China (Grant code: 2020YFC2008700), and XL was supported by Guangdong Basic and Applied Basic Research Foundation (Grant code: 2020A1515111134) and China Postdoctoral Science Foundation (Grant code: 2021M692215).

## Conflict of Interest

The authors declare that the research was conducted in the absence of any commercial or financial relationships that could be construed as a potential conflict of interest.

## Publisher's Note

All claims expressed in this article are solely those of the authors and do not necessarily represent those of their affiliated organizations, or those of the publisher, the editors and the reviewers. Any product that may be evaluated in this article, or claim that may be made by its manufacturer, is not guaranteed or endorsed by the publisher.

## Abbreviations

LS: Longshi Scale
BI: Barthel Index
mRS: modified Rankin Scale
ADL: Activities of Daily Living
MMSE: Mini-mental State Examination
QRL: Quicker Recovery Line
ROC: receiver operating characteristic
AUC: area under the curve
CI: confidence interval.

## References

[1] Stinear CM Lang CE Zeiler S Byblow WD. Advances and challenges in stroke rehabilitation. Lancet Neurol. (2020) 19:348–60. 10.1016/S1474-4422(19)30415-6 32004440

[2] Lee SY Kim DY Sohn MK Lee J Lee SG Shin YI. Determining the cut-off score for the Modified Barthel Index and the Modified Rankin Scale for assessment of functional independence and residual disability after stroke. PLoS ONE. (2020) 15:e0226324. 10.1371/journal.pone.0226324 31995563 PMC6988933

[3] Gao Y Wang Y Li D Zhao J Dong Z Zhou J. Disability assessment in stroke: Relationship among the pictorial-based Longshi Scale, the Barthel Index, and the modified Rankin Scale. Clin Rehab. (2020) 35:606–13. 10.1177/0269215520975922 33401949

[4] Liu F Tsang RC Zhou J Zhou M Zha F Long J. Relationship of Barthel Index and its Short Form with the Modified Rankin Scale in acute stroke patients. J Stroke Cerebrov Dis. (2020) 29:105033. 10.1016/j.jstrokecerebrovasdis.2020.105033 32807445

[5] Banks JL Marotta CA. Outcomes validity and reliability of the modified Rankin scale: implications for stroke clinical trials: a literature review and synthesis. Stroke. (2007) 38:1091–6. 10.1161/01.STR.0000258355.23810.c6 17272767

[6] Middleton S Dale S Cheung NW Cadilhac DA Grimshaw JM Levi C. Nurse-initiated acute stroke care in emergency departments. Stroke. (2019) 50:1346–55. 10.1161/STROKEAHA.118.020701 31092163

[7] Harrison JK. McArthur KS, Fau-Quinn TJ, Quinn TJ. Assessment scales in stroke: clinimetric and clinical considerations. Clin Interven Aging. (2013) 8:201–11. 10.2147/CIA.S32405 23440256 PMC3578502

[8] Matsuda T Iwagami MA-O Suzuki T Jin XA-O Watanabe TA-O Tamiya N. Correlation between the Barthel Index and care need levels in the Japanese long-term care insurance system. Geriatr Gerontol Int. (2019) 19:1186–7. 10.1111/ggi.13777 31746527

[9] Quinn TJ Langhorne P Fau - Stott DJ Stott DJ. Barthel index for stroke trials: development, properties, and application. Stroke. (2011) 42:1146–51. 10.1161/STROKEAHA.110.598540 21372310

[10] MacIsaac RL Ali M Taylor-Rowan M Rodgers H Lees KR Quinn TJ. Use of a 3-item short-form version of the barthel index for use in stroke: systematic review and external validation. Stroke. (2017) 48:618–23. 10.1161/STROKEAHA.116.014789 28154094

[11] Sulter G Steen C De Keyser J. Use of the Barthel Index and Modified Rankin Scale in acute stroke trials. Stroke. (1999) 30:1538–41. 10.1161/01.STR.30.8.1538 10436097

[12] Zhou M Liu X Zha F Liu F Zhou J Huang M. Stroke outcome assessment: Optimizing cutoff scores for the Longshi Scale, modified Rankin Scale and Barthel Index. PLoS ONE. (2021) 16:e0251103. 10.1371/journal.pone.0251103 33984006 PMC8118543

[13] Wang Y Guo S Zheng J Wang QM Zhang YA-O Liang Z. User testing of the psychometric properties of pictorial-based disability assessment Longshi Scale by healthcare professionals and non-professionals: a Chinese study in Shenzhen. Clin Rehabil. (2019) 33:1479–91. 10.1177/0269215519846543 31081365

[14] Assessment of self-care abilities in daily life for persons with disability. China: National Standards Committee and Ministry of Civil Affairs of the People's Republic of China. (2019). p. 1–11.

[15] Kokotailo RA Hill MD. Coding of stroke and stroke risk factors using international classification of diseases, revisions 9 and 10. Stroke. (2005) 36:1776–81. 10.1161/01.STR.0000174293.17959.a1 16020772

[16] Van Heugten CM Walton L Hentschel U. Can we forget the mini-mental state examination? A systematic review of the validity of cognitive screening instruments within one month after stroke. Clinical Rehabilitation. (2015) 29:694–704. 10.1177/0269215514553012 25381346

[17] Yilanda. Quicker Recovery Line. China (2022). Available online at: http://www.yilanda.cn/.

[18] Hung MC Hsieh CL Hwang JS Jeng JS Wang JD. Estimation of the long-term care needs of stroke patients by integrating functional disability and survival. PLoS ONE. (2013) 8:e75605. 10.1371/journal.pone.0075605 24124500 PMC3790845

[19] Akobeng AK. Understanding diagnostic tests 3: Receiver operating characteristic curves. Acta Paediatr. (2007) 96:644–7. 10.1111/j.1651-2227.2006.00178.x 17376185

[20] Uyttenboogaart M Luijckx GJ Vroomen PC Stewart RE De Keyser J. Measuring disability in stroke: relationship between the modified Rankin scale and the Barthel index. J Neurol. (2007) 254:1113–7. 10.1007/s00415-007-0646-0 17668259

[21] Sarker SJ Rudd AG Douiri A Wolfe CD. Comparison of 2 extended activities of daily living scales with the Barthel Index and predictors of their outcomes: cohort study within the South London Stroke Register (SLSR). Stroke. (2012) 43:1362–9. 10.1161/STROKEAHA.111.645234 22461336

[22] Nakao S Takata S Uemura H Kashihara M Osawa T Komatsu K. Relationship between Barthel Index scores during the acute phase of rehabilitation and subsequent ADL in stroke patients. J Med Invest. (2010) 57:81–8. 10.2152/jmi.57.81 20299746

[23] Hacke W Albers G Al-Rawi Y Bogousslavsky J Davalos A Eliasziw M. The Desmoteplase in Acute Ischemic Stroke Trial (DIAS): a phase II MRI-based 9-hour window acute stroke thrombolysis trial with intravenous desmoteplase. Stroke. (2005) 36:66–73. 10.1161/01.STR.0000149938.08731.2c 15569863

[24] Uyttenboogaart M Stewart RE Vroomen PC De Keyser J Luijckx GJ. Optimizing cutoff scores for the Barthel index and the modified Rankin scale for defining outcome in acute stroke trials. Stroke. (2005) 36:1984–7. 10.1161/01.STR.0000177872.87960.61 16081854

[25] Kwon S Hartzema AG Duncan PW Min-Lai S. Disability measures in stroke: relationship among the Barthel Index, the Functional Independence Measure, and the Modified Rankin Scale. Stroke. (2004) 35:918–23. 10.1161/01.STR.0000119385.56094.32 14976324

[26] Wang Y Li S Pan W Xiao P Zhang J Wang QM. Evaluation of the disability assessment Longshi scale: a multicenter study. J Int Med Res. (2020) 48:300060520934299. 10.1177/0300060520934299 32696703 PMC7376381

[27] Cohen ME Marino RJ. The tools of disability outcomes research functional status measures. Arch Phys Med Rehab. (2000) 81:21–9. 10.1053/apmr.2000.20620 11128901

[28] Dromerick AW Edwards DF Diringer MN. Sensitivity to changes in disability after stroke: a comparison of four scales useful in clinical trials. J Rehab Res Develop. (2003) 40:1–8. 10.1682/JRRD.2003.01.0001 15150715

[29] Yuan J Wang Y Hu W Bruno A. The reliability and validity of a novel Chinese version simplified modified Rankin scale questionnaire (2011). BMC Neurol. (2020) 20:1–5. 10.1186/s12883-020-01708-1 32268886 PMC7140377

